# Deep Multimodal Learning From MRI and Clinical Data for Early Prediction of Neurodevelopmental Deficits in Very Preterm Infants

**DOI:** 10.3389/fnins.2021.753033

**Published:** 2021-10-05

**Authors:** Lili He, Hailong Li, Ming Chen, Jinghua Wang, Mekibib Altaye, Jonathan R. Dillman, Nehal A. Parikh

**Affiliations:** ^1^Imaging Research Center, Cincinnati Children's Hospital Medical Center, Cincinnati, OH, United States; ^2^Department of Radiology, Cincinnati Children's Hospital Medical Center, Cincinnati, OH, United States; ^3^Department of Radiology, University of Cincinnati College of Medicine, Cincinnati, OH, United States; ^4^Department of Electronic Engineering and Computing Systems, University of Cincinnati, Cincinnati, OH, United States; ^5^Biostatistics and Epidemiology, Cincinnati Children's Hospital Medical Center, Cincinnati, OH, United States; ^6^The Perinatal Institute, Cincinnati Children's Hospital Medical Center, Cincinnati, OH, United States; ^7^Department of Pediatrics, University of Cincinnati College of Medicine, Cincinnati, OH, United States

**Keywords:** deep learning, neurodevelopment, very preterm infants, MRI, resting state functional MRI, diffusion tensor imaging, brain connectome, diffuse white matter abnormality

## Abstract

The prevalence of disabled survivors of prematurity has increased dramatically in the past 3 decades. These survivors, especially, very preterm infants (VPIs), born ≤ 32 weeks gestational age, are at high risk for neurodevelopmental impairments. Early and clinically effective personalized prediction of outcomes, which forms the basis for early treatment decisions, is urgently needed during the peak neuroplasticity window—the first couple of years after birth—for at-risk infants, when intervention is likely to be most effective. Advances in MRI enable the noninvasive visualization of infants' brains through acquired multimodal images, which are more informative than unimodal MRI data by providing complementary/supplementary depicting of brain tissue characteristics and pathology. Thus, analyzing quantitative multimodal MRI features affords unique opportunities to study early postnatal brain development and neurodevelopmental outcome prediction in VPIs. In this study, we investigated the predictive power of multimodal MRI data, including T2-weighted anatomical MRI, diffusion tensor imaging, resting-state functional MRI, and clinical data for the prediction of neurodevelopmental deficits. We hypothesize that integrating multimodal MRI and clinical data improves the prediction over using each individual data modality. Employing the aforementioned multimodal data, we proposed novel end-to-end deep multimodal models to predict neurodevelopmental (i.e., cognitive, language, and motor) deficits independently at 2 years corrected age. We found that the proposed models can predict cognitive, language, and motor deficits at 2 years corrected age with an accuracy of 88.4, 87.2, and 86.7%, respectively, significantly better than using individual data modalities. This current study can be considered as proof-of-concept. A larger study with external validation is important to validate our approach to further assess its clinical utility and overall generalizability.

## Introduction

With the continuing high incidence of preterm births (about 380,000 in 2018) (Martin et al., 2019) and improving survival rates (exceeding 90%) (Blencowe et al., 2012) in the United States, the prevalence of disabled survivors of prematurity has increased dramatically. These survivors, especially, very preterm infants (VPIs), born ≤ 32 weeks gestational age (GA), are at high risk for cognitive deficits and other neurodevelopmental disorders, thereby increasing their risk for poor educational, health, and social outcomes (Jarjour, 2015). Efforts to target interventions to prevent and/or treat neurodevelopmental sequelae are hampered by our current inability to diagnose or predict risk of disabilities before the age of 3–5 years (Nordhov et al., 2010; Kwon et al., 2014). The imminent challenge lies in early identification of infants who are at the greatest risk for developing later disorders at an individual level. Early and clinically effective personalized prediction of outcomes, which forms the basis for early treatment decisions, is urgently needed during the peak neuroplasticity window—the first couple of years after birth—for at-risk infants, when intervention is likely to be most effective (Johnston, 2009).

Advances in MRI enable the noninvasive visualization of infants' brains through acquired multi-modal images. Research supports the findings that brain imaging features are modulated by genetic (Thompson et al., 2001), non-genetic biological (Hackman and Farah, 2009), and environmental (May, 2011) influences, and therefore show high variability among subjects. Such variability can potentially provide valuable information for personalized prognosis based on the characteristics of individual patients (Valizadeh et al., 2018). Brain anatomical features have been recently extended to the prognostication of neurodevelopmental impairments (cognitive, motor, working memory, and language), autism spectrum disorder (ASD), and attention deficit hyperactivity disorder (ADHD) (Boardman et al., 2010; Thompson et al., 2014; Chaddad et al., 2017). We have externally validated our findings (He and Parikh, 2013; Li et al., 2019; Parikh et al., 2020) that objectively-diagnosed diffuse white matter abnormality (DWMA) at term equivalent age is an independent predictor of cognitive and language development in VPIs. In addition, brain connectivity patterns are formed during early brain development and reshaped in cases of prematurity or perinatal brain injury (Cao et al., 2017). Brain connectome studies have revealed microstructural alterations in cognition and motor tracts that correlate with poorer cognitive and motor performance (Thompson et al., 2014; Rogers et al., 2016). Atypical functional connectivity has been reported in children who develop adverse cognitive, language and motor outcomes (He and Parikh, 2015; Gozdas et al., 2018; He et al., 2020). Multimodal MRI data are more informative than unimodal MRI data by providing complementary/supplementary depicting of how brain tissue characteristics and their pathology information are segregated and integrated. Therefore, accurately analyzing quantitative multimodal MRI features affords unique opportunities to study early postnatal brain development and neurodevelopmental outcome prediction in preterm infants (Thompson et al., 2016). Through this, we may gain a better understanding of how an individual brain's organizational changes influence cognitive, language, and motor functions.

Although, it is easy to understand, how to endow machines with capabilities to perceive patients through comprehensive information from multiple imaging or other data modalities is still an open question. The feature representations from different modalities originally locate in unequal subspaces, resulting that similar feature representations may be associated with completely different semantics. Therefore, the biggest challenge is how to project heterogeneous features into a common space, where the multimodal data with similar semantics will be represented by similar features (Rasiwasia et al., 2010; Guo et al., 2019). In the computer vision domain, studies have been conducted to address this problem in various applications, such as, video description and classification (Liu et al., 2016), event detection (Wu et al., 2014), cross-modal retrieval and translation (Qi and Peng, 2018; Wu et al., 2018), image caption (Xu et al., 2015), and text-to-image synthesis (Reed et al., 2016). In light of these existing works, and with recent advances in deep learning techniques (Hjelm et al., 2014; Plis et al., 2014; Mostapha and Styner, 2019), we propose to encode each unimodal representation, and then fuse the encoded unimodal features.

Unlike most published studies that describe unimodal MRI data (Kawahara et al., 2017; Moeskops et al., 2017; Girault et al., 2019; He et al., 2020; Saha et al., 2020), in this paper, we employed multimodal MRI and proposed deep multimodal learning models. We hypothesize that integrating multimodal MRI and clinical data improves early prediction of cognitive, language, and motor deficits independently, at 2 years corrected age in VPIs over using each individual data modality. By doing so, the proposed prediction model is capable of analyzing different types of inputs by fusing different neural networks. Specifically, the different model inputs, which were all collected at term-equivalent age, include: (1) structural brain connectome data from diffusion tensor imaging (DTI); (2) functional brain connectome data from resting-state functional MRI (rs-fMRI) connectome data; (3) DWMA quantified from anatomical T2-weighted images; and (4) perinatal clinical data. The fusion technique used here is a concatenation of the four encoded feature vectors, which is then used as an input to fully-connected layers before the network outputs its prediction. The resulting classification system is a deep multimodal learning model, an automated prognostic system that uses four types of data as inputs to determine at term-equivalent age whether or not an individual VPI is at high risk of developing moderate or more severe cognitive, language, and/or motor deficits and to predict individual standardized neurodevelopmental scores (on the Bayley Scales of Infant and Toddler Development, Third Edition (Bayley III) (Bayley, 2009) Cognitive, Language, and Motor subtest scores) at 2 years corrected age.

The main contributions of our work are highlighted as follows: (1) We proposed end-to-end deep multimodal learning models that incorporate features from multimodal MRI (anatomical, DTI, and rs-fMRI) and clinical data; (2) We demonstrated that the application of deep multimodal learning to analyze high-dimensional objectively-quantified anatomical and connectome features may detect brain structural and functional abnormalities and tissue pathology that are not readily visible to the naked eye, thereby facilitating risk stratification; (3) We unwrapped and identified discriminative MRI and clinical features used by the proposed models to make predictions. Such discriminative feature identification will generate greater trust in the prognostic models and enhanced pathophysiologic understanding.

## Methods and Materials

### Subjects and MRI Acquisition

The Institutional Review Boards of the Nationwide Children's Hospital (NCH) and Cincinnati Children's Hospital Medical Center (CCHMC) approved this study, and written parental informed consent was obtained for every subject. This study has been carried out in accordance with The Code of Ethics of the World Medical Association. This study included 261 prospectively recruited VPIs from five Cincinnati Ohio neonatal intensive care units (NICUs) as cohort I (for unsupervised model pre-training), and 108 VPIs from four Columbus area/Central Ohio NICUs as cohort II (for supervised model fine-tuning). All subjects were scanned during natural sleep without the use of any sedation after being fed and swaddled. Infants with congenital structural central nervous system anomalies (e.g., Dandy-Walker, encephalocele, diffuse calcifications, and meningomyelocele) or congenital chromosomal abnormalities known to be associated with neurodevelopmental impairments were excluded.

Subjects in the Cohort I were scanned at 39–44 weeks postmenstrual age (PMA) on a 3T MRI scanner (Ingenia, Philips Healthcare, Best, The Netherlands) at CCHMC using a 32-channel head coil. Anatomical scans were conducted with a 2D T2-weighted fast spin-echo sequence. Functional MRI data were conducted using multi-band rs-fMRI (multi-band factor = 3). Diffusion MRI data were collected using single-shot echo planar imaging (EPI). Detailed acquisition parameters are listed in Supplementary Table 1.

All cohort II subjects were scanned at 38–43 weeks PMA on a 3T MRI scanner (Skyra; Siemens Healthcare) at NCH using a 32-channel head coil. Anatomical scans were conducted with a 2D T2-weighted fast spin-echo sequence. Functional MRI data were collected using single-band/multi-band rs-fMRI (multi-band factor = 3). Diffusion MRI data were collected using single-shot EPI. Detailed acquisition parameters are also listed in Supplementary Table 1.

### Clinical Features and Neurodevelopmental Assessments

For each VPI, 72 a priori defined and prospectively collected perinatal clinical features were retrieved (Supplementary Table 2). Clinical features related to five overarching domains, including: (1) maternal demographics (e.g., mothers age, gravida, parity, mother's highest educational level, etc.); (2) pregnancy complications (e.g., diabetes, hypertension, hypothyroidism, etc.); (3) labor and delivery (e.g., rupture of membranes, antenatal steroids, magnesium administration, etc.); (4) neonatal information at birth (e.g., sex, gestational age, birth weight, etc.); and (5) medical history (e.g., oxygen or positive pressure support, surfactant administration, pneumothorax, sepsis, bronchopulmonary dysplasia, etc.).

The Bayley III Cognitive, Language, and Motor subtest scores [each standardized on a scale of 40–160, with a mean of 100 and standard deviation (SD) of 15] served as the primary neurodevelopmental outcome measures. We dichotomized the VPIs using Bayley-III score of 90 into those high-risk (≤90) vs. low-risk (>90) for neurodevelopmental deficits.

### DWMA Quantification

We quantified DWMA using our published objective algorithm (He and Parikh, 2013). Briefly, brain tissue segmentation (white matter, gray matter, and cerebrospinal fluid) was achieved by unified segmentation on T2-weighted images with spatial priors obtained from a neonatal probabilistic atlas (Shi et al., 2011). We considered voxels with signal intensity values greater than α standard deviation above the mean of cerebral (white + gray matter) tissues to be DWMA. Volume of DWMA was calculated as the product of voxel volume and total number of voxels in the detected DWMA region. We determined the normalized volume of DWMA by dividing DWMA volume by total cerebral white matter volume. The optimal α may be different for different cohort MRI data acquired with different imaging protocols (He and Parikh, 2013, 2015; Li et al., 2019; Parikh et al., 2020). Instead of determining one single optimal α value, in this work, to take advantage of the strength of feature integration, we defined a DWMA feature vector which contained a series of DWMA volumes that were obtained by varying the threshold α from 1.4 to 2.0 with increment of 0.1. To control inter-subject variability, we also include the volume of white matter, gray matter, and CSF as confounders into the DWMA feature vector.

### Structural Connectome Quantification

We preprocessed DTI data with a pipeline involving skull stripping, registration, head motion, and eddy current artifacts correction using FMRIB Software Library (FSL, Oxford University, UK) (Woolrich et al., 2009). We conducted diffusion tensor reconstruction based on a linear least-square fitting algorithm and brain fiber tracking based on a deterministic tracking algorithm in the subject's native space using Diffusion Toolkit/TrackVis (Wang et al., 2007). We harmonized fractional anisotropy maps using a batch-effect correction algorithm ComBat (Fortin et al., 2017) to remove undesirable variabilities caused by different acquisition parameters. The brain was parcellated into 90 regions of interest (ROIs) according to a neonatal anatomical template (Shi et al., 2011), forming the nodes of the individual structural networks. Structural connectivity map (i.e., 90 × 90 network adjacency matrix symmetric about the diagonal), were constructed using the UCLA Multimodal Connectivity Package (Bassett et al., 2011). Each entry in the structural connectome map represents the brain structural connectivity between each pair of ROIs, which was calculated as the mean fractional anisotropy of each voxel intersecting the tract and then averaged over all tracts between the two nodes.

### Functional Connectome Quantification

We performed rs-fMRI preprocessing using previously validated pipelines (Pogribna et al., 2014; He and Parikh, 2016), to (1) Reorient all acquired scans with anterior commissure (AC)—posterior commissure (PC) line; (2) Remove non-brain parts of the image; (3) Correct motion artifact by aligning each time point's frame to the middle frame, and estimate corresponding six motion parameters [three translation (displacement) and three rotation parameters]; (4) Register both rs-fMRI and structural T2-weighted images to be in the same “standard space” [a neonatal brain atlas (Shi et al., 2011)]; (5) Regress out the mean time courses of cerebral white matter, ventricles, and whole brain and their derivatives; as well as six motion parameters and their derivatives and squares (Power et al., 2014); (6) Improve signal-to-noise ratio and ameliorate the effects of functional misalignments across subjects (Lowe and Sorenson, 1997) using spatial smoothing with isotropic Gaussian filter with 6 mm kernel; and (7) remove the lowest and highest temporal drifts in the data *via* band-pass filtering (0.008 < *f* <0.09 Hz; Hallquist et al., 2013). We then parcellated the brain into 223 ROIs according to a neonatal functional template (Shi et al., 2017), forming the nodes of the individual brain functional networks. We extracted rs-fMRI time series from each ROI, then computed the functional connectivity as the correlation between the time series of each pair of ROIs. This resulted in a functional connectome map (i.e., 223 × 223 network adjacency matrix symmetric about the diagonal). All above operations were conducted using FMRIB Software Library (FSL, Oxford University, UK), Statistical Parametric Mapping software (SPM, University College London, UK; Friston, 1994) and functional connectivity toolbox (CONN) (Whitfield-Gabrieli and Nieto-Castanon, 2012). We also conducted connectome map harmonization using the ComBat algorithm (Fortin et al., 2017).

### Data Augmentation and Balancing

We conducted data augmentation and balancing on the training data to enable a robust model training. A challenge in the proposed supervised model training is the relatively small number of infants at high-risk compared to those at low-risk. Imbalanced datasets can severely affect the model's learning ability (Haixiang et al., 2017). In such cases, the deep learning models may become majority class classifiers, i.e., they fail to learn the concepts of the minority class. To overcome this challenge, we employed a data balancing and augmentation method (Kawahara et al., 2017), which uses neighborhood samples to create artificial minority samples. By synthetically generating more samples of the minority class, the classifiers are able to broaden their decision regions for the minority class. Specifically, similar to a prior work (Kawahara et al., 2017), we first categorized supervised training dataset into five bins according to a VPI's Bayley-III subtest score (<70, 70–79, 80–89, 90–100, and >100). We randomly selected a sample (i.e., functional, or structural connectivity data) in a bin with the fewest samples and searched for *k* nearest neighbors for the given sample based on Euclidean distance. Assuming that the selected sample is *x*_0_, and its associated neighbors are [*x*_1_, … *x*_*i*_, …, *x*_*k*_], a synthetic data *x*_*s*_ is generated by: *x*_*s*_ =_β_0_*x*0_ + β_1_*x*_1_+…β_*i*_*x*_*i*_+…β_*k*_*x*_*k*_, where β_*i*_ is a random weight, and $\overset{k}{\underset{i=0}{\sum }}\beta_{i}=1$. The corresponding Bayley-III score *y*_*s*_ was generated in the same way. We repeatedly generated synthetic samples for each bin until the numbers of training samples in all bins were equal. This process was also repeated until the number of training samples reached 10 times that of the original training dataset. Importantly, the synthetic data were only used for model training, but not for testing.

### Model Design

**Figure 1:**
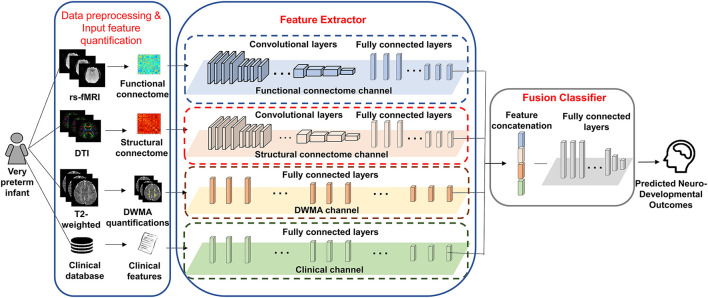
A deep multimodal learning model consists of feature extractor and fusion classifier, for the prediction of neurodevelopmental (cognitive, language, and motor) deficits using MRI and clinical data.

We proposed deep multimodal learning models for the early prediction of cognitive, language, and motor deficits using multimodal MRI and clinical data (Figure 1). We have presented how imaging and clinical data were acquired and preprocessed, as well as how multimodal MRI features were quantified in subsections (Subjects and MRI Acquisition, Clinical Features and Neurodevelopmental Assessments, DWMA Quantification, Structural Connectome Quantification, Functional Connectome Quantification, and Data Augmentation and Balancing). Each of our proposed models contain a feature extractor and a fusion classifer. The feature extractor has four parallel channels to extract discriminative high-level functional and structural connectivity, DWMA, and clinical features out of high-dimensional input data, respectively. Both functional and structural connectivity channels have the same network architecture. It consists of 16 convolutional layers and 5 pooling layers adopted from the pre-trained VGG-19 model (Simonyan and Zisserman, 2014), followed by fully connected blocks. Since the feature dimensions of the DWMA and clinical data are not high, both DWMA and clinical channels only consist of fully connected blocks, without pre-trained VGG-19 layers for the feature dimensionality reduction. Each fully connected block contains a fully connected layer, a batch normalization, and a dropout layer. The dropout layer is a regularization technique that randomly selects a certain ratio of neurons and ignores them during training (Srivastava et al., 2014). The “dropped-out” neurons do not contribute to the feedforward process, and the weights of these neurons are not updated in backpropagation. Dropout regularization helps avoid model overfitting. Batch normalization solves the internal covariate shift problem (Ioffe and Szegedy, 2015). Similar to feature scaling, batch normalization works to adjust, and scale hidden unit shifts across hidden layers. Batch normalization also speeds up the training process when handling a large number of features. Finally, we design a fusion classier to integrate the discriminative information from all extracted high-level imaging and clinical features using a fully connected layer with one output neuron. We conduct the outcome classification using a softmax function and outcome regression using a linear function.

### Model Training and Optimization

Deep learning models generally require training on large datasets to achieve good performance while our annotated dataset for the target tasks (i.e., prediction of cognitive, language, and motor deficits) is relatively small. To address this issue, we utilized both supervised and unsupervised transfer learning approaches. In particular, the VGG-19 (Simonyan and Zisserman, 2014) layers described above were pretrained with supervision using ImageNet database (~1.2 million images). The weights of these layers were fixed and reused in our model. The weights of all other neural network layers were first pretrained without supervision using a relatively large unannotated VPI data from cohort I. These weights were finally retrained and fine-tuned in a supervised fashion using annotated VPI data from cohort II for outcome classification/regression. The mechanism behind this rationale is that we can repurpose models developed for other tasks ulitizing a large dataset to ultimately improve the performance and generalizbility of our proposed models as well as decrease the amount of data needed for model training.

Specifically, given *m* training samples in the cohort I, $\left[{\text{X}}_{f}^{i},{\text{X}}_{s}^{i},{\text{x}}_{d}^{i},{\text{x}}_{c}^{i}\right],\;i\;\in \;[1,m]$ are the input data of the *i-th* sample without label, where ${\text{X}}_{f}^{i}$ is a two-dimensional adjacency matrix (i.e., 223 × 223) of functional connectivity; ${\text{X}}_{s}^{i}$ is a two-dimensional adjacency matrix (i.e., 90 × 90) of structural connectivity; ${\text{x}}_{d}^{i}$ is the one-dimensional vector (i.e., 1 × 11) of DWMA measures; and ${\text{x}}_{c}^{i}$ is a one-dimensional vector (i.e., 1 × 72) of clinical data. As mentioned above, we first utilized pretrained VGG-19 layers to extract high-level morphological features of adjacency matrix from both functional and structural connectivity. The outputs of VGG-19 layers are flattened as one-dimensional vectors (i.e., 1 × *k*) and denoted by $H\left({\text{X}}_{f}^{i}\right)$ and $H\left({\text{X}}_{s}^{i}\right)$.

Next, to mitigate the issue of mismatch between ImageNet database and the small annotated VPI dataset in cohort II, we continued to perform an unsupervised transfer learning using the relatively large unannotated VPI dataset from cohort I. Except for VGG-19 layers, we pretrained the weights of all other neural network layers of both functional and structural connectivity channels without supervision. We pretrained the fully connected layers of both functional and structural connectivity channels using an unsupervised learning strategy. We constructed a stacked sparse autoencoder (SSAE) for the fully connected layers. A rectified linear unit (ReLU) activation function was used in hidden nodes, and a sigmoid unit was chosen in the output layer. For each brain connectivity channel, we minimized the mean squared error loss function:


$$\begin{array}{l} L=\;-\frac{1}{m}\overset{m}{\underset{i=1}{\sum }}\overset{k}{\underset{j=1}{\sum }}{\left({H\left({\text{X}}^{i}\right)}^{j}-{\hat{H}\left({\text{X}}^{i}\right)}^{j}\right)}^{2} \end{array}$$

where ${\hat{H}\left({\text{X}}^{i}\right)}^{j}$is the reconstructed functional or structural input ${H\left({\text{X}}^{i}\right)}^{j}$from *j-th* neuron of the SSAE. A mini-batch Adam algorithm (Kingma and Ba, 2014) was selected to minimize the loss function. The learning rate was selected from empirical values [0.001, 0.01, 0.1, and 0.5]. Batch size was chosen using (Hackman and Farah, 2009; Johnston, 2009; Nordhov et al., 2010; Blencowe et al., 2012). Total number of epochs was 50. These hyperparameters were optimized based on validation data during model training/validation before model testing.

With these pretrained fully connected layers, we continued to retrain and fine-tune the whole model using a supervised training strategy using annotated VPI data from cohort II. Assume that there are *n* training samples in cohort II, and $\left[{\text{X}}_{f}^{i},{\text{X}}_{s}^{i},{\text{x}}_{d}^{i},{\text{x}}_{c}^{i}\right],\;i\;\in \;[1,n]$ are the input data of the *i-th* sample with label/score *y*^*i*^, *i* ∈ [1, *n***]** (i.e., high risk vs. low risk of developing cognitive, language, or motor deficits). For the classification task, we fine-tuned the fully connected layers and fusion classifier of the model by minimizing cross-entropy loss function as:


$$\begin{array}{ll} L=-\frac{1}{n}\overset{n}{\underset{i=1}{\sum }}y^{i}\log\left(p\left(y^{i}|H\left({\text{X}}_{f}^{i}\right),H\left({\text{X}}_{s}^{i}\right),{\text{x}}_{d}^{i},\;{\text{x}}_{c}^{i}\right)\right)\\ + & \left(1-y^{i}\right)\log\left(1-p\left(y^{i}|H\left({\text{X}}_{f}^{i}\right),H\left({\text{X}}_{s}^{i}\right),{\text{x}}_{d}^{i},\;{\text{x}}_{c}^{i}\right)\right) \end{array}$$

where $p\left(y^{i}|H\left({\text{X}}_{f}^{i}\right),H\left({\text{X}}_{s}^{i}\right),{\text{x}}_{d}^{i},\;{\text{x}}_{c}^{i}\right)\;$is the output of the fusion classifier, i.e., the probability of subject *i* being classified as the label *y*^*i*^. For the score regression task, we applied a linear unit at the end of the model and optimized the mean absolute error loss function as follows:


$$\begin{array}{l} L=\frac{1}{n}\overset{n}{\underset{i=1}{\sum }}|y^{i}-{ŷ}^{i}\left(H\left({\text{X}}_{f}^{i}\right),H\left({\text{X}}_{s}^{i}\right),{\text{x}}_{d}^{i},\;{\text{x}}_{c}^{i}\right)| \end{array}$$

where ${ŷ}^{i}\left(H\left({\text{X}}_{f}^{i}\right),H\left({\text{X}}_{s}^{i}\right),{\text{x}}_{d}^{i},\;{\text{x}}_{c}^{i}\right)$ is the predicted output of the linear unit of the model, i.e., the predicted score. The mini-batch Adam algorithm was also used in the supervised learning. Training hyperparameters are listed in Table 2. To accelerate the model convergence, we applied an adaptive gradient update decay parameter (e.g., learning rate/maximal epoch). We used an early stop mechanism, which would cease the optimization process when multiple consecutive epochs returned the same validation loss errors.

With the fixed optimized pre-trained VGG19, our model architecture optimization focuses on the determination of the optimal number of fully connected layers and the optimal number of neurons at each layer. During the model training and validation, we tried the numbers of layers with empirical values from 1 to 4 in increments of 1; and we tried the numbers of neurons at each layer with empirical values from: 2^*n*^, *n* ∈ [3, 4, 5, 6]. For each architecture setting, we ran 2-fold validations multiple times. According to the optimal validation performance, we set the optimal modal architecture (Table 1). The final training hyperparameters are listed in Table 2.

**Table 1:** Architecture of the proposed deep multimodal learning model.

**Layer index**	**Functional connectivity channel**		**Structural connectivity channel**		**DWMA channel**		**Clinical channel**	
(1)	Conv2D, ReLU (fixed)	64@3 × 3	Conv2D, ReLU (fixed)	64@3 × 3	FC (trainable)	64	FC (trainable)	64
(2)	Conv2D, ReLU (fixed)	64@3 × 3	Conv2D, ReLU (fixed)	64@3 × 3	Batch Norm.	N/A	Batch Norm.	N/A
(3)	Maxpooling 2D	2	Maxpooling 2D	2	Dropout	0.2	Dropout	0.2
(4)	Conv2D, ReLU (fixed)	128@3 × 3	Conv2D, ReLU (fixed)	128@3 × 3	FC (trainable)	16	FC (trainable)	16
(5)	Conv2D, ReLU (fixed)	128@3 × 3	Conv2D, ReLU (fixed)	128@3 × 3	Batch Norm.	N/A	Batch Norm.	N/A
(6)	Maxpooling 2D	2	Maxpooling 2D	2	Dropout	0.2	Dropout	0.2
(7)	Conv2D, ReLU (fixed)	256@3 × 3	Conv2D, ReLU (fixed)	256@3 × 3				
(8)	Conv2D, ReLU (fixed)	256@3 × 3	Conv2D, ReLU (fixed)	256@3 × 3				
(9)	Conv2D, ReLU (fixed)	256@3 × 3	Conv2D, ReLU (fixed)	256@3 × 3				
(10)	Conv2D, ReLU (fixed)	256@3 × 3	Conv2D, ReLU (fixed)	256@3 × 3				
(11)	Maxpooling 2D	2	Maxpooling 2D	2				
(12)	Conv2D, ReLU (fixed)	512@3 × 3	Conv2D, ReLU (fixed)	512@3 × 3				
(13)	Conv2D, ReLU (fixed)	512@3 × 3	Conv2D, ReLU (fixed)	512@3 × 3				
(14)	Conv2D, ReLU (fixed)	512@3 × 3	Conv2D, ReLU (fixed)	512@3 × 3				
(15)	Conv2D, ReLU (fixed)	512@3 × 3	Conv2D, ReLU (fixed)	512@3 × 3				
(16)	Maxpooling 2D	2	Maxpooling 2D	2				
(17)	Conv2D, ReLU (fixed)	512@3 × 3	Conv2D, ReLU (fixed)	512@3 × 3				
(18)	Conv2D, ReLU (fixed)	512@3 × 3	Conv2D, ReLU (fixed)	512@3 × 3				
(19)	Conv2D, ReLU (fixed)	512@3 × 3	Conv2D, ReLU (fixed)	512@3 × 3				
(20)	Conv2D, ReLU (fixed)	512@3 × 3	Conv2D, ReLU (fixed)	512@3 × 3				
(21)	Maxpooling 2D	2	Maxpooling 2D	2				
(22)	FC (trainable)	64	FC (trainable)	64				
(23)	Batch Norm.	N/A	Batch Norm.	N/A				
(24)	Dropout	0.2	Dropout	0.2				
(25)	FC (trainable)	16	FC (trainable)	16				
(26)	Batch Norm.	N/A	Batch Norm.	N/A				
(27)	Dropout	0.2	Dropout	0.2				
**Fusion classifier**								
(28)	FC (trainable)				8			
(29)	Batch Norm.				N/A			
(30)	Softmax				2			

*Conv2D, 2D convolutional layer; Maxpooling 2D, 2D Maxpooling layer; Batch Norm. Batch Normalization layer; FC, Fully connected layer*.

**Table 2:** Hyperparameters for the unsupervised and supervised training.

**Hyperparameters**	**Value**
High-risk neurodevelopmental deficits	>0
Low-risk neurodevelopmental deficits	0
Batch size	4
Dropout rate	0.2
Total number of epochs	50
Learning rate	0.01

The proposed model development was implemented using Python 3.7.4, Keras (version: 2.1.6) with TensorFlow (version 1.14) backend on a computer workstation (256 GB RAM, 2 GPUs, Nvidia GTX1080 Ti).

### Most Discriminative Feature Identification

To unravel and illuminate the proposed deep multimodal learning models' predictive feature identification process and to generate greater trust in the models, we first adopted a feature ranking approach (Olden and Jackson, 2002) for one dimensional input of deep learning models to identify the most predictive clinical and DWMA risk factors. Specifically, we calculated the partial derivatives of the softmax output with respect to the clinical and DWMA features. For the softmax output (i.e., neurodevelopmental deficit) *s*, the partial derivatives $\frac{\partial s}{\partial f_{i}^{c}}\;$and $\frac{\partial s}{\partial f_{j}^{d}}\;$, where $f_{i}^{c}$ is the *i*th clinical feature and $f_{j}^{d}$ is the *j*th DWMA features, are computed for individual clinical and DWMA features. A higher absolute value of the partial derivative of $\frac{\partial s}{\partial f_{i}^{c}}\;$and $\frac{\partial s}{\partial f_{j}^{d}}\;$indicates a higher level of the importance for neurodevelopmental deficit prediction *s*.

We then implemented gradient-weighted class activation mapping (Grad-CAM) algorithm (Selvaraju et al., 2017), which was designed for two dimensional image input of deep learning models, to highlight both discriminative structural and functional brain connectivity in brain connectome maps (i.e., adjacency matrices). The Grad-CAM produces a coarse localization map highlighting predictive brain connectivities in the adjacency matrix by using gradient information of the last convolutional layer of the structural and functional channels (refer to Figure 1 and Table 1). Specifically, we first computed the gradient of the softmax output *s* respect to the *k*th 2D feature map *A* of the last convolutional layer by $\frac{\partial s}{\partial A_{ij}^{k}}$, where *i, j* ∈ [1, *m*], and *m* is the size of feature maps. Then, we obtained the weights of feature maps as $\alpha_{k}=GAP\left(\frac{\partial s}{\partial A_{ij}^{k}}\right)$, where *GAP*(^*^) is the global average pooling function. The heatmap of Grad-CAM was obtained by calculating the ReLU activation of the weighted combination of feature maps as: $H=ReLU\left(\underset{k}{\sum }\alpha_{k}A^{k}\right)$. The heatmap *H* was then normalized to [0, 1] and rescaled to the same size as adjacency matrices of structural and functional connectome. A higher value within *H* indicates a higher level of the importance for neurodevelopmental deficit prediction *s*.

### Model Validation

To evaluate the performance of the risk stratification (i.e., two-class classification), we calculated balanced accuracy, sensitivity, specificity, and area under the receiver operator characteristics curve (AUC). To evaluate the performance of the Bayley III score prediction (i.e., regression), we reported Pearson's correlation coefficient (*r*), mean absolute error (MAE) and standard deviation of absolute error (SD of AE). We conducted nested five-fold cross-validation. In each iteration, the entire cohort II was divided into training data (60%), validation data (20%), and testing data (20%). Model optimization was conducted based on validating data without seeing testing data. We conducted this process for five iterations until all the cohort had been tested once. We then computed the performance across all five iterations. To test the reproducibility of the model, we repeated such five-fold cross-validation experiment 50 times and reported mean and standard deviation (SD).

### Statistical Analysis

Continuous demographic data and model performance metrics (described in the section Model Validation) were summarized as means and SDs, and categorical demographic data were summarized as counts and percentages. The two-sided Student's *t*-test (continuous data) and Chi-squared test (categorical data) were used to assess demographic characteristic differences between groups. The two-sided Student's *t*-test was also utilized to compare the model performances of using different feature sets. A *p* < 0.05 was considered statistically significant. Analyses were performed with the statistical package of Matlab 2019b (MathWorks, Natick MA, United States).

## Results

### Subjects

After data quality control, excluding the data with largely incomplete brain coverage, high movement peaks, ghosting, incomplete imaging scans, and other scanner artifacts, we included 257 of 261 VPIs (mean (SD) GA at birth 29.3 (2.5) weeks; PMA at scan 42.7 (1.3) weeks; 111 (43.2%) male) without Bayley III assessments (cohort I), and 72 of 108 VPIs (mean (SD) GA at birth 28.3 (2.4) weeks; PMA at scan 40.3 (0.5) weeks; 41 (56.9%) male) with Bayley III assessments (cohort II). For all three neurodevelopmental (cognitive language, and motor) deficits prediction tasks, PMA was not significantly different between high-risk and low-risk groups. As expected, GA and birth weight were significantly different between the high-risk and low-risk groups. Additional demographic data for cohort II subjects with neurodevelopmental assessments at 2 years corrected age is listed in Table 3.

**Table 3:** Demographic information of cohort II subjects with neurodevelopmental assessments at 2 years corrected age.

	**Cognitive deficit**		
	**Low risk** **(*N* = 47)**	**High risk** **(*N* = 25)**	***p*-value**
PMA at early MRI in weeks; median (range)	40.3 (39.4–42.0)	40.1 (39.3–41.4)	0.5
GA in weeks; median (range)	29.1 (24.0–31.9)	28.0 (23.7–31.4)	0.04
Birth weight in grams; median (range)	1165 (570–2,340)	855 (510–1,755)	0.02
Sex; number of males (percentage)	27 (57.4%)	14 (56.0%)	0.906
	**Language deficit**		
	**Low risk** **(*****N*** **=** **47)**	**High risk** **(*****N*** **=** **25)**	***p*** **-value**
PMA at early MRI in weeks; median (range)	40.3 (39.4–42.0)	40.1 (39.3–41.3)	0.14
GA in weeks; median (range)	29.7 (24.0–31.8)	26.8 (23.7–31.0)	0.0004
Birth weight in grams; median (range)	1285 (570–2,340)	855 (510–1,400)	<0.0001
Sex; number of males (percentage)	26 (55.3%)	15 (60.0%)	0.7025
	**Motor deficit**		
	**Low risk** **(*****N*** **=** **30)**	**High risk** **(*****N*** **=** **42)**	***p*** **-value**
PMA at early MRI in weeks; median (range)	40.1 (39.4–41.3)	40.3 (39.3–42.0)	0.1
GA in weeks; median (range)	29.9 (25.1–31.9)	27.7 (23.7–31.4)	0.0003
Birth weight in grams; median (range)	1325 (715–1,900)	878 (510–2,340)	0.0004
Sex; number of males (percentage)	18 (60.0%)	23 (54.7%)	0.6581

*PMA, postmenstrual age; GA, gestational age*.

### Cognitive Deficit Prediction

We tested the model performance of classifying VPIs into high- vs. low-risk group and predicting actual Bayley III Cognitive scores (i.e., continuous scale) using only clinical, functional connectome, structural connectome, and DWMA data alone; and then using combined features. As shown in Table 4, our model was able to correctly identify high-risk infants for cognitive deficits with a mean (SD) AUC of 0.87 ± 0.05 and the Pearson's correlation coefficient *r* between the predicted and actual Bayley III Cognitive scores of 0.62 ± 0.04 (*p* < 0.0001) using the combined clinical and multi-modal MRI data. This was significantly greater than individually using only, (1) clinical data [AUC = 0.74 ± 0.05 (*p* < 0.0001) and *r* = 0.34 ± 0.06 (*p* < 0.0001)]; (2) functional connectome data [AUC = 0.74 ± 0.05 (*p* < 0.0001) and *r* = 0.34 ± 0.07 (*p* < 0.0001)]; (3) structural connectome data [AUC = 0.81 ± 0.06 (*p* < 0.0001) and *r* = 0.44 ± 0.05 (*p* < 0.0001)]; and (4) DWMA data [AUC = 0.74 ± 0.05 (*p* < 0.0001) and *r* = 0.39 ± 0.04 (*p* < 0.0001)]. These support our hypothesis that integrating multimodal MRI and clinical data improves early prediction of cognitive deficits at 2 years corrected age in VPIs over using individual data modalities.

**Table 4:** Performance comparison shows that our proposed deep multimodal learning model that uses combined feature sets (i.e., functional connectome + structural connectome + clinical data + DWMA) obtained at term-equivalent age outperforms each individual feature set for early identification of very preterm infants at high-risk for cognitive deficits and predicting their actual Bayley III Cognitive scores at 2 years corrected age.

**Features**	**Cognitive deficit risk stratification**				**Cognitive Bayley III score prediction**		
	**Accuracy (%)**	**Sensitivity (%)**	**Specificity (%)**	**AUC**	***r***	**MAE**	**SD of AE**
Functional connectivity	72.0 ± 4.6	63.4 ± 7.7	80.5 ± 6.0	0.74 ± 0.05	0.34 ± 0.07	19.5	12.6
Structural connectivity	79.9 ± 4.1	73.5 ± 5.7	86.4 ± 6.0	0.81 ± 0.06	0.44 ± 0.05	13.3	11.8
Clinical features	77.3 ± 4.2	73.8 ± 5.1	82.3 ± 6.2	0.77 ± 0.05	0.41 ± 0.05	15.5	12.6
DWMA	80.6 ± 5.7	78.3 ± 4.8	82.8 ± 5.0	0.74 ± 0.05	0.39 ± 0.04	14.9	13.1
**Combined**	**88.4** **±** **3.7**	**83.4** **±** **6.5**	**90.3** **±** **5.2**	**0.87** **±** **0.05**	**0.62** **±** **0.04**	**11.7**	**8.6**

### Language Deficit Prediction

We next evaluated the model performance for language deficit risk stratification and Bayley III Language score prediction using individual and combined feature sets (Table 5). The model using the functional connectome alone achieved the lowest balanced accuracy of 74.8 ± 3.9%, while the one using DWMA data alone had the lowest Pearson's correlation coefficient *r* of 0.39 ± 0.06. The deep multimodal learning model using combined features achieved the highest performance for risk stratification with a balanced accuracy of 87.2 ± 5.3% and AUC of 0.85 ± 0.04. These were significantly higher than the second highest balanced accuracy of 78.4 ± 4.2% (*p* < 0.0001) using DWMA alone, and the second highest AUC of 0.78 ± 0.04 (*p* < 0.0001) using clinical features alone. The deep multimodal learning model achieved a Pearson's correlation coefficient *r* of 0.63 ± 0.04 between the predicted and actual Bayley III language scores, significantly higher than the one using functional connectome (*p* < 0.0001), structural connectome (*p* < 0.0001), clinical data (*p* < 0.0001), and DWMA data (*p* < 0.0001). The results support our hypothesis that integrating multimodal MRI and clinical data improves early prediction of language deficits at 2 years corrected age in VPIs over using individual data modalities.

**Table 5:** Performance comparison shows that our proposed deep multimodal learning model using combined feature sets (i.e., functional connectome + structural connectome + clinical data + DWMA) obtained at term-equivalent age outperforms each individual feature set for early identification of very preterm infants at high-risk for language deficits and predicting their actual Bayley III Language scores at 2 years corrected age.

**Features**	**Language deficit risk stratification**				**Language Bayley III score prediction**		
	**Accuracy (%)**	**Sensitivity (%)**	**Specificity (%)**	**AUC**	***r***	**MAE**	**SD of AE**
Functional Connectivity	74.8 ± 3.9	71.3 ± 5.4	78.2 ± 6.4	0.76 ± 0.04	0.44 ± 0.06	14.4	12.1
Structural Connectivity	76.2 ± 4.7	74.1 ± 7.2	78.2 ± 6.1	0.75 ± 0.05	0.48 ± 0.05	12.9	11.1
Clinical features	75.9 ± 4.2	74.2 ± 6.5	81.3 ± 4.2	0.78 ± 0.04	0.40 ± 0.06	13.7	10.4
DWMA	78.4 ± 4.2	75.3 ± 5.7	81.5 ± 5.9	0.78 ± 0.05	0.39 ± 0.06	15.4	10.2
**Combined**	**87.2** **±** **5.3**	**83.6** **±** **6.4**	**89.6** **±** **6.1**	**0.85** **±** **0.04**	**0.63** **±** **0.04**	**10.5**	**8.2**

*DWMA, diffuse white matter abnormality; r, Pearson's correlation coefficient; MAE, mean absolute error; SD of AE, standard deviation of absolute error*.

### Motor Deficit Prediction

Table 6 demonstrates the model performance for classifying high- vs. low-risk motor deficit group and predicting actual Bayley III Motor scores using individual and combined feature sets. The model using combined features was able to correctly identify high-risk VPIs for motor deficits with an AUC of 0.85 ± 0.06, significantly better than using functional connectome (0.71 ± 0.05; *p* < 0.0001), structural connectome (0.75 ± 0.05; *p* < 0.0001), clinical data (0.75 ± 0.06; *p* < 0.0001), and DWMA data (0.76 ± 0.05; *p* < 0.0001). This model also achieved the highest Person's correlation coefficient *r* of 0.63 ± 0.05 (*p* < 0.0001). This was significantly greater than using functional connectome data with a *r* of 0.38 ± 0.06 (*p* < 0.0001), structural connectome data with a *r* of 0.45 ± 0.07 (*p* < 0.0001), clinical data with a *r* of 0.41 ± 0.06 (*p* < 0.0001), and DWMA data with a *r* of 0.38 ± 0.05 (*p* < 0.0001). These support our hypothesis that integrating multimodal MRI and clinical data improves early prediction of motor deficits at 2 years corrected age in VPIs over using individual data modalities.

**Table 6:** Performance comparison shows that our proposed deep multimodal learning model using combined feature sets (i.e., functional connectome + structural connectome + clinical data + DWMA) obtained at term-equivalent age outperforms each individual feature set for early identification of very preterm infants at high-risk for motor deficits and predicting their actual Bayley III Motor scores at 2 years corrected age.

**Features**	**Motor deficit risk stratification**				**Motor Bayley III score prediction**		
	**Accuracy (%)**	**Sensitivity (%)**	**Specificity (%)**	**AUC**	***r***	**MAE**	**SD of AE**
Functional connectivity	68.6 ± 4.8	70.9 ± 6.3	66.2 ± 8.1	0.71 ± 0.05	0.38 ± 0.06	15.9	12.5
Structural connectivity	74.1 ± 5.2	71.3 ± 8.2	77.0 ± 6.1	0.75 ± 0.06	0.45 ± 0.07	13.7	12.4
Clinical features	75.4 ± 5.3	73.4 ± 5.7	78.2 ± 6.3	0.75 ± 0.06	0.41 ± 0.06	13.8	10.7
DWMA	77.1 ± 4.7	80.7 ± 4.8	73.4 ± 6.4	0.76 ± 0.05	0.38 ± 0.05	14.5	11.1
**Combined**	**86.7** **±** **5.2**	**87.6** **±** **5.8**	**82.5** **±** **4.9**	**0.85** **±** **0.06**	**0.63** **±** **0.05**	**11.6**	**9.2**

*DWMA, diffuse white matter abnormality; r, Pearson's correlation coefficient; MAE, mean absolute error; SD of AE, standard deviation of absolute error*.

### Most Discriminative Feature Identification

**Figure 2:**
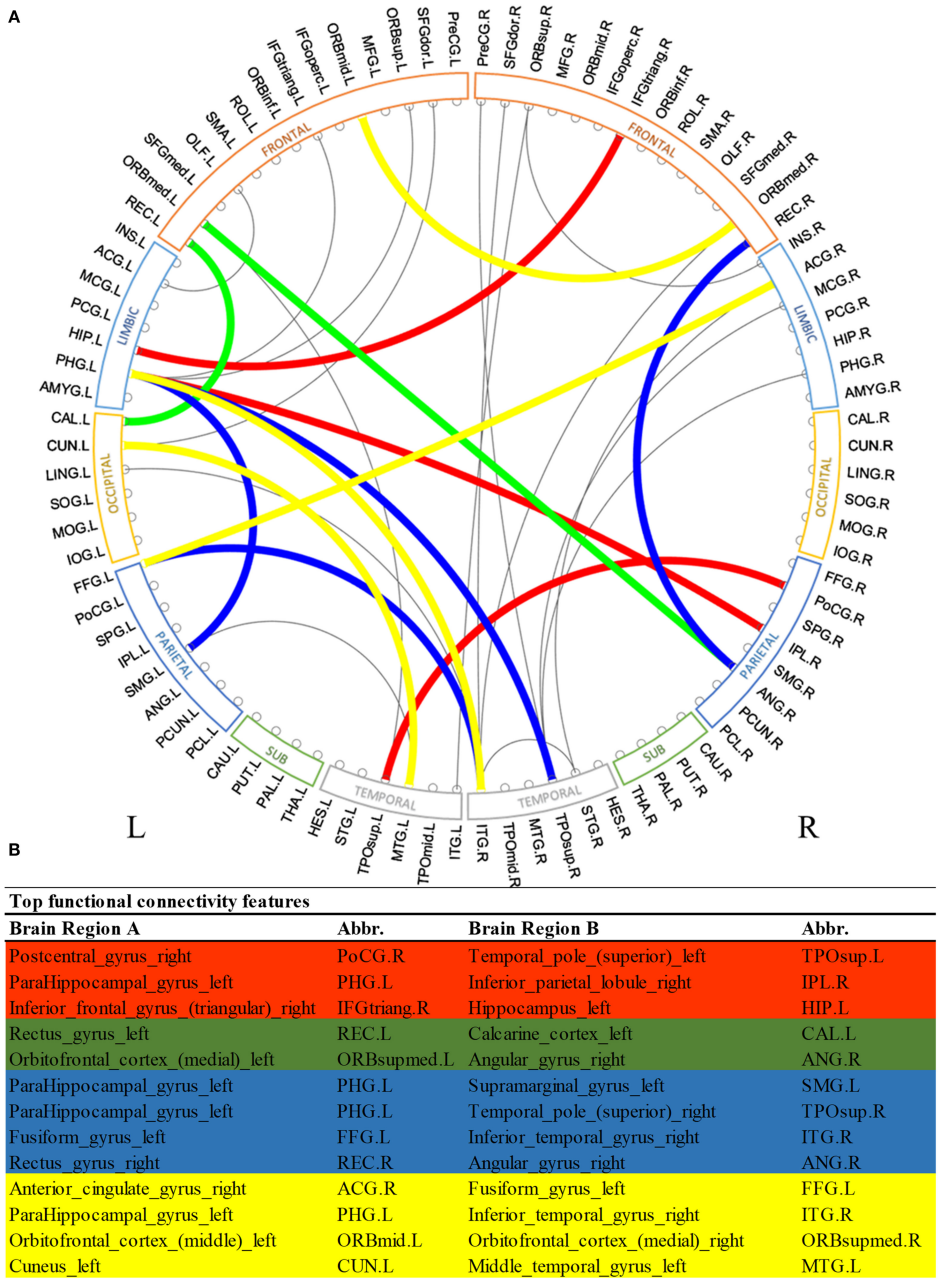
Top discriminative region-to-region functional connections for early prediction of cognitive, language, and motor deficits. **(A)** circos plot visualization; **(B)** Full names and abbreviations table. Three common connections were identified to be important for the prediction of all three deficits (red); five common connections were identified to be predictive of both cognitive and language deficits (red and green); seven common connections were identified to be predictive of both language and motor deficits (red and blue); and seven common connections were identified to be predictive of both cognitive and motor deficits (red and yellow).

Figure 2 shows the most discriminative region-to-region *functional* connections ranked by the proposed deep multimodal learning model for the prediction of cognitive, language, and motor deficits. Among 13 functional connections discriminative for at least two deficits, 8% are within the right hemisphere and 23% are within the left hemisphere only. Interhemispheric connections account for 69% of top discriminative connections. More detailed predictive functional connections to the individual deficits are shown in Supplementary Figures 1–3. Functional brain connections contributing to the prediction of all three deficits span frontal, limbic, occipital, temporal, and parietal lobes.

**Figure 3:**
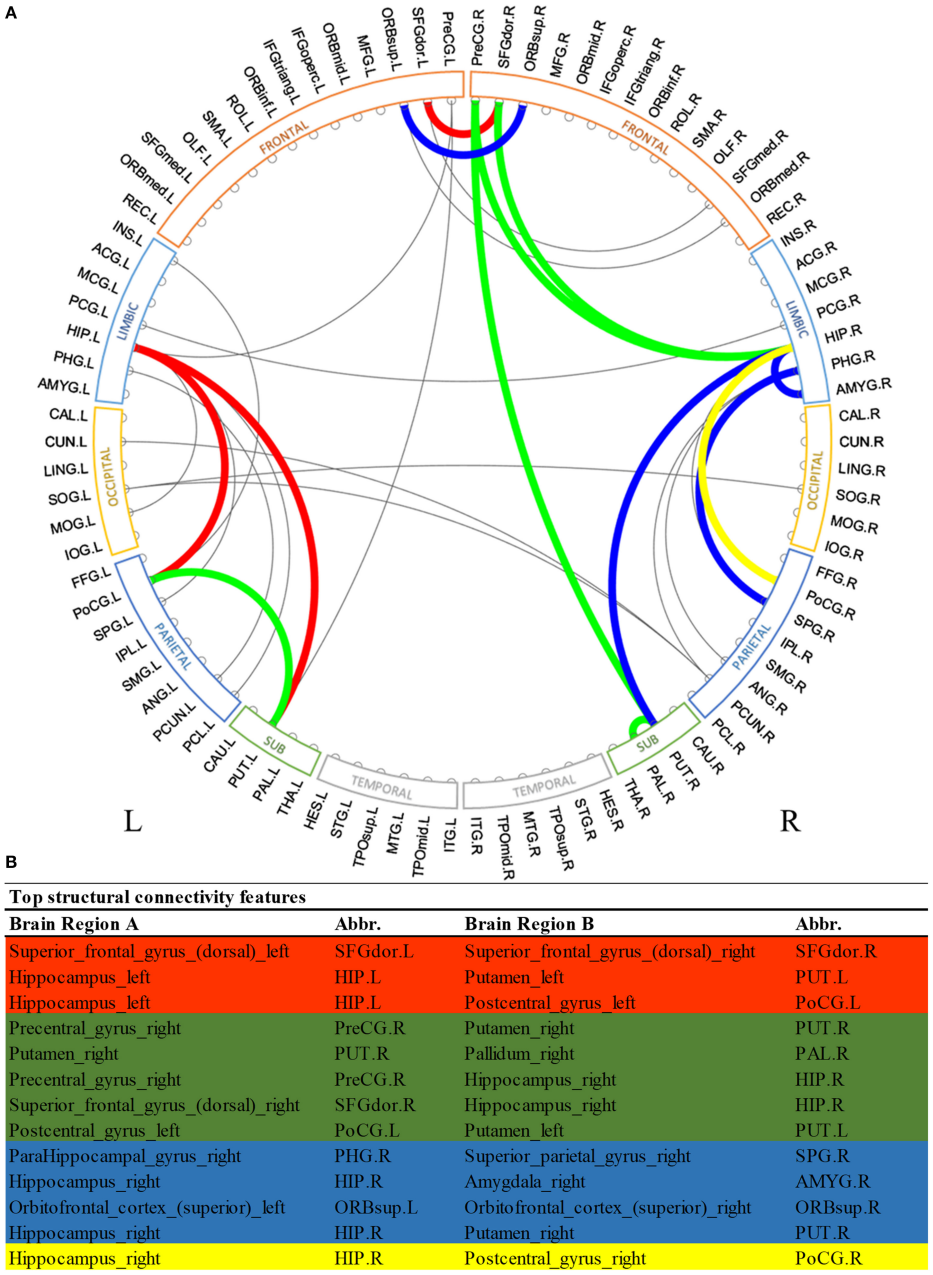
Top discriminative region-to-region structural connections for early prediction of cognitive, language, and motor deficits. **(A)** circos plot visualization; **(B)** Full names and abbreviations table. Three common connections were identified to be important for the prediction of all three deficits (red); eight common connections were identified to be predictive of both cognitive and language deficits (red and green); seven common connections were identified to be predictive of both language and motor deficits (red and blue); and four common connections were identified to be predictive of both cognitive and motor deficits (red and yellow).

Similarly, Figure 3 shows the most predictive *structural* connections ranked by the proposed deep multimodal learning model for the prediction of all three deficits. Among 13 structural connections discriminative for at least two deficits, 62% are within the right hemisphere and 23% are within the left hemisphere. Fifteen percent of top discriminative connections are interhemispheric connections. Structural brain connections contributing to the prediction of all three deficits focus on frontal, limbic, and parietal lobes, as well as subcortical gray nuclei. More detailed predictive structural connections to the individual deficits are shown in Supplementary Figures 4–6.

Table 7 shows the discriminative clinical features ranked by our deep multimodal learning model for the prediction of all three neurodevelopmental (cognitive, language, and motor) deficits. As expected, several well-known neurodevelopment-relevant clinical features were repeatedly selected by the model as discriminative features for all three prediction tasks, such as mother's highest educational level, infant positive pressure respiratory therapy, head circumference at birth, birth weight, and gestational age at birth. Among 11 severity levels of DWMA feature, we found that threshold α = 1.8 DWMA feature was ranked as the most predictive DWMA feature for all three prediction tasks.

**Table 7:** Top discriminative clinical features for early prediction of cognitive, language, and motor deficits.

**Cognition**		**Language**		**Motor**	
**Features**	**Weights**	**Features**	**Weights**	**Features**	**Weights**
Head circumference at birth (cm)	0.9	Birth weight (grams)	1.0	Mothers age	1.1
Mothers age	0.9	Head circumference at birth (cm)	0.8	Birth weight (grams)	1.0
Gestational age at birth (total weeks)	0.8	Gestational age at birth (weeks)	0.8	Parenteral alimentation (days)	0.9
Parenteral alimentation (days)	0.8	Highest education level	0.8	Maternal smoking status	0.9
Pulmonary hypertension history	0.8	Mothers age	0.8	PRBC transfusions	0.9
Birth weight (grams)	0.8	PRBC transfusions	0.7	Total time on oxygen therapy (days)	0.8
Fertility treatment	0.7	Birth length (cm)	0.7	Head circumference at birth (cm)	0.8
Birth length (cm)	0.7	Pulmonary hypertension history	0.7	Birth length (cm)	0.8
Gestational age at birth (weeks)	0.7	CS history before 35 weeks PMA	0.7	Patent ductus arteriosus history	0.8
Highest education level	0.7	Referral to child protective services	0.7	Pulmonary hypertension history	0.8
Pulmonary hemorrhage	0.6	Total time on oxygen therapy (days)	0.7	Ventilation therapy	0.8
Ventilation therapy	0.6	Maternal annual income	0.6	Respiratory support type at 36-week	0.8
Necrotizing enterocolitis	0.6	CS history at or after 35 weeks PMA	0.6	Pulmonary hemorrhage	0.7
Iron supplementation	0.6	Parenteral alimentation (days)	0.6	Gestational age at birth (weeks)	0.7
GI surgery that resulted in short gut	0.6	Retinopathy of prematurity exam	0.6	Iron supplementation	0.7
PRBC transfusions	0.6	Pulmonary hemorrhage	0.6	GI surgery that resulted in short gut	0.7
Total time on oxygen therapy (days)	0.6	Placental pathology	0.6	Pneumothorax	0.7
Hyperthyroidism	0.6	Birth hospital	0.6	Total number of days on CPAP	0.7
Surfactant	0.6	Total number of days on CPAP	0.5	Necrotizing enterocolitis	0.7
Seizure history	0.5	Chorioamnionitis	0.5	Infections history	0.7

## Discussion

### Brain Connectome Data Are Predictive of Neurodevelopmental Deficits

There is an increasing consensus that human brain can be modeled as a complex network both at a structural as well as functional level (Stam et al., 2016). Structural networks typically represent connection pathways corresponding to white matter tracks between pairs of brain regions, measuring white matter integrity. Functional networks represent magnitudes of temporal cross-correlations between blood-oxygen-level dependent (BOLD) signals, measuring coupling strength. Neurodevelopmental deficits can be understood as dysconnectivity syndromes, therefore the quantifications of the abnormal structural and functional network using graph theory may enable neurodevelopmental prognosis. In VPIs, we have previously established correlations of later neurodevelopmental outcomes with at term obtained functional connectivity features derived from rs-fMRI (Gozdas et al., 2018); and structural connectivity features derived from DTI (Chen et al., 2020). In this work, our results showed both structural and functional connectivity features obtained at term-equivalent age are predictive of abnormal cognitive, language, and motor outcomes at 2 years corrected age. Our results also suggest that the predictive power of structural connectivity features is stronger than functional connectivity features. The significant performance improvement supports our hypothesis that integrating multimodal MRI and clinical data improves early prediction of cognitive, language, and motor deficits independently, at 2 years corrected age in VPIs over using each individual data modality.

Recent advances in deep learning techniques, based on artificial neural networks (ANN), have made it possible to extract physiologically meaningful features and reveal new discriminative information from high dimensional MRI data (Hjelm et al., 2014; Plis et al., 2014; Mostapha and Styner, 2019). Applications of deep learning to analyze high-dimensional objectively-quantified connectome features derived from DTI, and rs-fMRI data may detect brain structural and functional abnormalities and tissue pathologies that are not readily visible to the human eye, thereby facilitating risk stratification (Kassner and Thornhill, 2010; Mostapha and Styner, 2019; Sahiner et al., 2019). There is a growing interest in developing deep learning approaches to predict a variety of brain disorders and neurodevelopmental deficits using MRI data (Wee et al., 2012; Kawahara et al., 2017; Gilmore et al., 2018; He et al., 2018; Heinsfeld et al., 2018; Girault et al., 2019; Saha et al., 2020). However, early prediction of neurodevelopmental deficits for preterm infants is a very challenging task. For example, Kawahara et al. (2017) developed a BrainNetCNN model to predict cognitive and motor developmental outcome scores from brain structural connectome with a Person's correlation coefficient *r* of 0.188 and 0.310, respectively. In another study, Saha et al. (2020) achieved a mean accuracy of 73% on predicting motor outcome in preterm infants by applying a CNN model on DTI data. Similarly, we previously developed a transfer learning neural network model using functional connectome data to predict cognitive outcome at 2 years of corrected age, achieved an accuracy of 70.6% (He et al., 2018). These studies using single modality data demonstrated that deep learning models were promising tools, but there is still a long way ahead. In the current work, we demonstrated that deep multimodal learning model is able to significantly improve prediction performance by integrating multiple data modalities. This facilitates the early prediction of neurodevelopmental deficits for preterm infants in the clinical setting using deep learning models and multimodal data.

### Potential Brain Connectome Biomarkers at Birth of Later Neurodevelopment

We observed multiple common functional brain connections, bridging brain regions within bilateral frontal lobe, left limbic system, left temporal lobe, and right parietal lobe, that significantly contributed to the prediction of all three neurodevelopmental deficits at 2 years corrected age (Figure 2). These regions serve important functions for language, sensory, motor, and cognitive function. For example, our proposed model identified the functional connection between the right postcentral gyrus and superior part of left temporal pole in all prediction tasks. The postcentral gyrus is located within the parietal lobe and is adjacent to the precentral gyrus of the frontal lobe (which was also selected). It is the primary somatosensory cortex and the main sensory receptive area (Hyvärinen and Poranen, 1978). On the other hand, the temporal pole is involved in high level semantic representation and socio-emotional processing (Olson et al., 2007). It is conceivable that the network between these brain regions is involved in cognitive, language, and motor functions as assessed by the Bayley III standardized tests at 2 years corrected age. Several other regions that are well-established hubs for these three core functions, such as the inferior temporal gyrus, inferior frontal gyrus, and cuneus were also identified as predictive biomarkers by our multimodal model. These results highlight the self-taught learning capability of the proposed deep multimodal learning model.

In terms of structural brain connectome, we also found multiple common connections that significantly contributed to decision-making of all three neurodevelopmental deficits at 2 years corrected age (Figure 3). Bilateral putamen regions were associated with some of these discriminative structural connections. Putamen is a critical subcortical nuclei that regulates movement and learning (de Jong et al., 2008). Significant microstructural or macrostructural alterations of putamen have been associated with neurodevelopmental and neurodegenerative disorders, including developmental language impairment (Lee et al., 2013), Parkinson's disease (Menke et al., 2009), and epilepsy (Keller et al., 2011; Gerdes et al., 2012). For example, Keller et al. (2011) demonstrated increased fractional anisotropy and decreased volume of the putamen region in patients with juvenile myoclonic epilepsy. Furthermore, the fractional anisotropy of putamen was showed to be significantly correlated with age in prior studies (Snook et al., 2005; Silk et al., 2009). This enables putamen to be a potential biomarker of human brain developmental trajectory. In another study, Fischi-Gómez et al. (2015) showed that decreased connectivity between basal ganglia (caudate, putamen, and globus pallidum combined) with frontal or parietal regions was associated with cognitive and emotional development in school age extremely preterm infants. We also previously demonstrated that the lenticular nucleus (combined putamen and globus pallidum) is ~15% smaller in extremely low birth weight infants as compared to full-term controls (Parikh et al., 2013). Apparently, our model took advantage of discriminative information embedded in the putamen-related structural connections for the neurodevelopmental prediction in this current work. Anatomically, the putamen is closely connected to the pallidum region. The short-range structural connection between putamen and pallidum within the right hemisphere was identified by our model to be predictive for both cognitive and language deficits. Our finding is consistent with several previous studies in non-VPI populations that highlighted the synchronization and dyssynchronization of putamen and pallidum (Cheruel et al., 1994; de Jong et al., 2008; Gooijers et al., 2016). Noteworthily, our model identified the structural connection between putamen and hippocampus within the left hemisphere for all neurodevelopmental deficits risk stratification, but only associated the mirror connection within the right hemisphere to language and motor deficits. It might be interesting to further investigate the mechanism behind such differences between structural connections linking putamen and hippocampus of left and right hemispheres.

The hippocampus was repeatedly identified by our models for all three prediction tasks using both brain functional and structural connectome data. The hippocampus is well-known for its primary role in organizing and storing information, and particularly in forming new memories (Kesner, 2007; Ekstrom and Ranganath, 2018). Prior studies reported that patients with mild Alzheimer's disease exhibited altered hippocampal activity on functional MRI during memory tasks (Small et al., 1999; Sperling, 2007). In a DTI study, mean diffusivity of the hippocampus was significantly associated with verbal memory performance (den Heijer et al., 2012). Our model appears to recognize the importance of hippocampus structurally and functionally. Our findings support the idea that the hippocampus plays a critical role in learning and cognition during early infancy (Beauchamp et al., 2008). These further indicate that our proposed deep multimodal learning model is capable of automatically learning and identifying neurologically meaningful functional and structural connectivity for prediction tasks of neurodevelopmental deficits. Intriguingly, the model identified multiple structural connections related to bilateral hippocampi, while it only recognized one functional connection associated with the hippocampus region within the left hemisphere of the brain. This may be due to fact that a multimodal integrative machine learning model tends to learn and utilize complementary features, instead of duplicated information. It is also notable that over half of the top discriminative functional connections were long-range connections across bilateral hemispheres, but only a small portion (15%) of structural connections were interhemispheric. Further investigation is needed to explore the influence of long-range functional connections and short-range structural connections on neurodevelopment of neonates.

### Identified Clinical and DWMA Predictors of Later Neurodevelopment

We identified several antepartum, intrapartum, and postnatal clinical factors that were predictive of one or more neurodevelopmental outcome at 2 years corrected age. Most of these factors have been shown in one or more prior studies to be predictive of such outcomes, including gestational age, birth growth parameters, duration of oxygen therapy/respiratory support and cognitive, language, and motor outcomes (Ambalavanan et al., 2012; Linsell et al., 2015, 2016; Parikh et al., 2020). These predictors that are consistent with prior research demonstrate the self-taught learning capability of our deep multimodal learning model on discovering useful knowledge from high dimensional big data. For DWMA features, the threshold α = 1.8 DWMA feature was ranked as the most predictive feature for all three prediction tasks. In a prior independent study, we also found that threshold α = 1.8 DWMA feature is significantly correlated with 2 years cognitive and language outcomes (Parikh et al., 2020). Importantly, the proposed deep multimodal learning model ranked these clinical predictors by simultaneously considering functional and structural connectome features. Thus, the rankings of these predictors do not necessarily reflect their individual predictive power on neurodevelopment. In other words, the most predictive variable in a univariable analysis may not be ranked as the top discriminative feature by our models.

### Related to Deep Multimodal Learning

It has been long recognized that the integration of multimodal features improves the performance of machine learning methods. Each feature modality has its own characteristic, which is different from others, leading to the complexity of heterogeneous data. Therefore, the key factor in multimodal fusion task is how to fill the heterogeneity gap of different modalities. For example, in this work, the problem is how to fuse heterogeneous features (i.e., very high dimensional structural and functional connectome data, as well as, low dimensional clinical and DWMA data) in a multimodal setting. In other words, how one solves the challenge of fusing high-dimensional and low-dimensional data will significantly impact the final results (Xu et al., 2016). If integrated directly, low-dimensional data would be completely overwhelmed by high-dimensional data. Instead, we proposed to encode each unimodal data *via* an independent neural network. By varying the architecture of the individual neural network, we reduced the dimensions of the high-dimensional data, and augmented or maintained the dimensions of the low-dimensional data. We then projected the encoded representations with equal dimensions into a shared semantic subspace, where the multimodal features/representations can be aggregated into a single feature/representation vector. Such learned vector is expected to fuse complementary and supplementary semantics from different modalities. The advantages of the multimodal learning strategy we proposed include: (1) convenience of fusing several modalities and (2) the shared common subspace tends to be modality-invariant, which is helpful for transferring knowledge from one modality to another (Guo et al., 2019).

### Study Limitations

This study has several limitations. First, though we have previously demonstrated that joint prediction of multiple neurodevelopmental deficits improves performance over independent prediction of each individual deficit (He et al., 2020), we opted to go with the latter approach in this work, since the training augmentation algorithm we used were not supported for multi-task label simulation. Second, the multimodal predictive feature identification was conducted based on the optimal multimodal neural network architecture rather than the optimal unimodal neural network. That is, the identified predictive unimodal features were constrained by the other modalities, therefore such feature identification schema cannot be used to infer the separated predictive features for each modality. Third, the current study is mainly about outcome prediction, more systematic statistical analysis will be needed to determine if brain connectome, DWMA or certain clinical risk factors are biomarkers for later neurodevelopment. Fourth, an atlas without the cerebellum was used for brain connectome quantification, however, functional and structural connections within the cerebellum may also be important for emerging functional outcomes. Finally, this current study should be considered as proof-of-concept due to the limited sample size. A larger population is necessary to test the model generalizability.

## Conclusion

We presented a novel deep multimodal learning framework integrating features derived from anatomical MRI, rs-fMRI, DTI, and clinical data obtained at term-equivalent age to predict Bayley-III developmental scores and identify very preterm infants at-high risk of developing cognitive, language, and motor deficits at 2 years corrected age. We demonstrated the value of multimodal MRI features as potential biomarkers for prediction of later neurodevelopmental deficits. We also reported a set of predictive functional and structural connections and clinical risk factors of neurodevelopmental deficits. A larger study with external validation is important to validate our approach to further assess its clinical utility and overall generalizability.

## Data Availability Statement

Requests to access the data sets used in this study should be directed to the corresponding author with a formal data sharing agreement and approval from the requesting researcher's local ethics committee. Requests to access these datasets should be directed to Lili He (lili.he@cchmc.org).

## Ethics Statement

The studies involving human participants were reviewed and approved by the Institutional Review Boards of the Nationwide Children's Hospital and Cincinnati Children's Hospital Medical Center. Written informed consent to participate in this study was provided by the participants' legal guardian/next of kin.

## Author Contributions

LH: conceptualization, methodology, validation, formal analysis, visualization, writing—original draft, and funding acquisition. HL: methodology, software, validation, formal analysis, visualization, and writing—review and editing. MC: software, validation, visualization, and formal analysis. JW, MA, and JD: validation and writing—review and editing. NP: conceptualization, resources, validation, writing—review, editing, and funding acquisition. All authors contributed to the article and approved the submitted version.

## Funding

This work was supported by the National Institutes of Health (R01-EB029944, R01-EB030582, R21-HD094085, R01-NS094200, and R01-NS096037). The funders played no role in the design, analysis, or presentation of the findings.

## Conflict of Interest

The authors declare that the research was conducted in the absence of any commercial or financial relationships that could be construed as a potential conflict of interest.

## Publisher's Note

All claims expressed in this article are solely those of the authors and do not necessarily represent those of their affiliated organizations, or those of the publisher, the editors and the reviewers. Any product that may be evaluated in this article, or claim that may be made by its manufacturer, is not guaranteed or endorsed by the publisher.
