# Supplementary material for: Deep Multimodal Learning From MRI and Clinical Data for Early Prediction of Neurodevelopmental Deficits in Very Preterm Infants
Source: Front Neurosci. 2021 Oct 5;15:753033. doi: 10.3389/fnins.2021.753033 (PMC8525883; doi:10.3389/fnins.2021.753033)
Supplement: Supplementary file 1 [file Data_Sheet_1.PDF]

Supplementary Material

1 Supplementary Figures

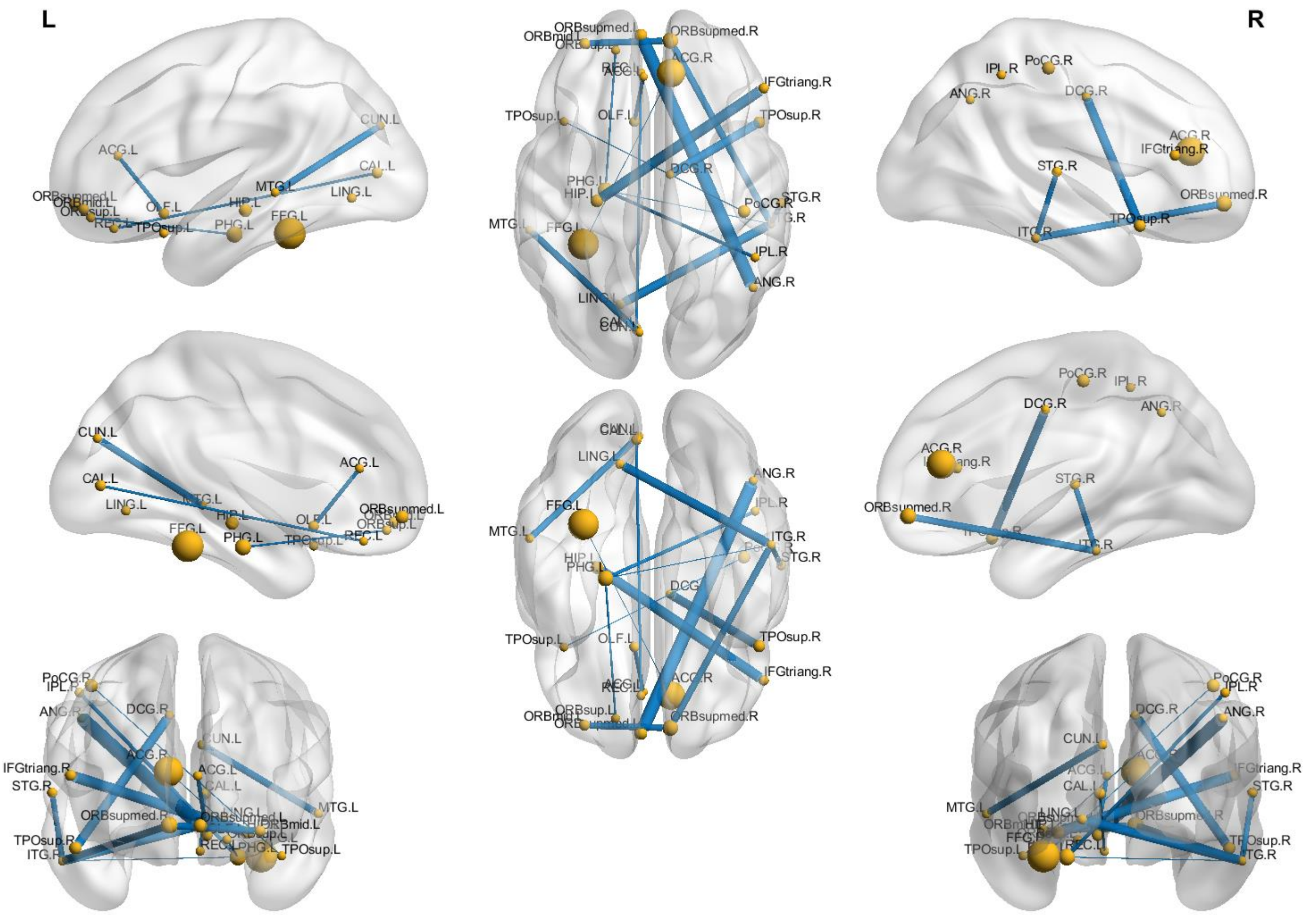

Supplemental figure 1. Top 15 discriminative brain structural connections on early prediction of cognitive deficits.

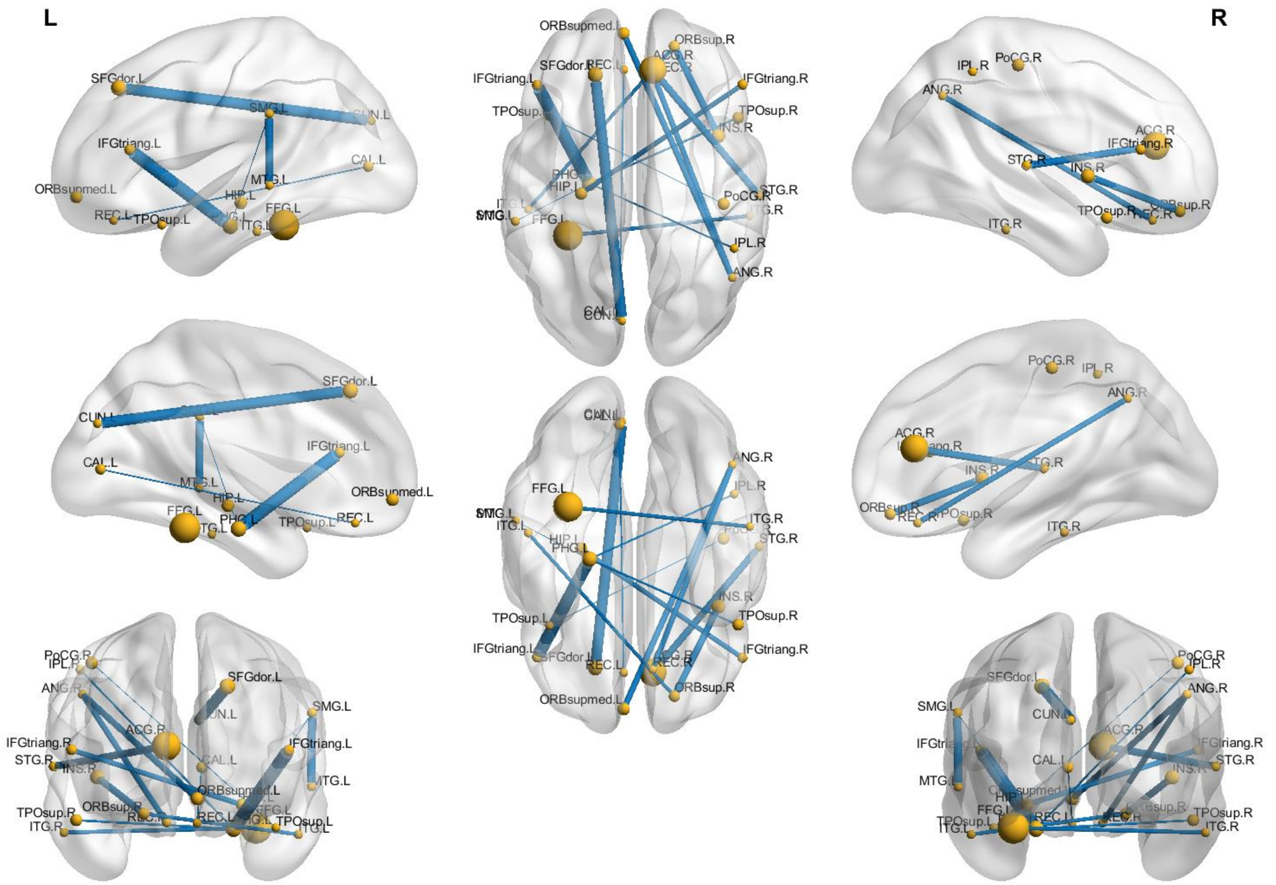

Supplemental figure 2. Top 15 discriminative brain structural connections on early prediction of language deficits.

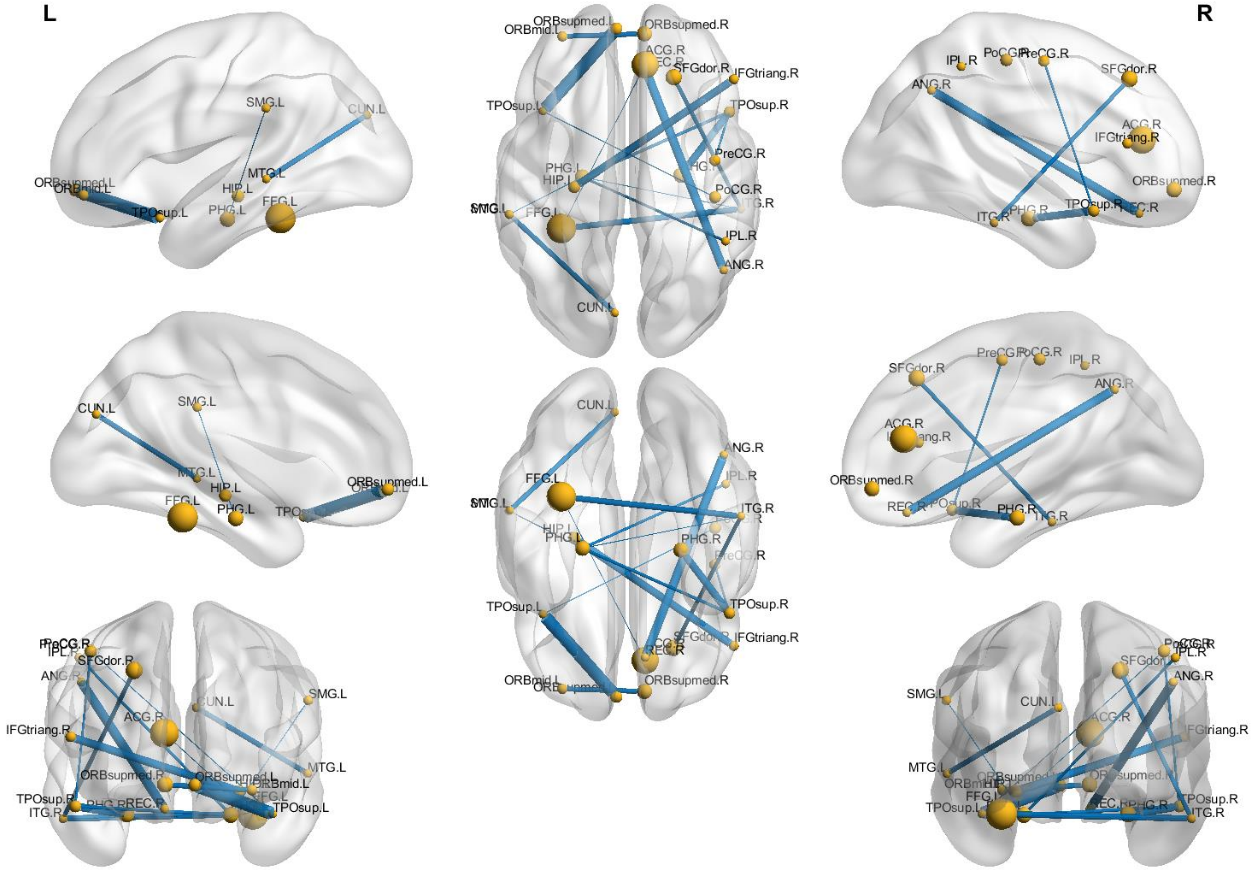

Supplemental figure 3. Top 15 discriminative brain structural connections on early prediction of motor deficits.

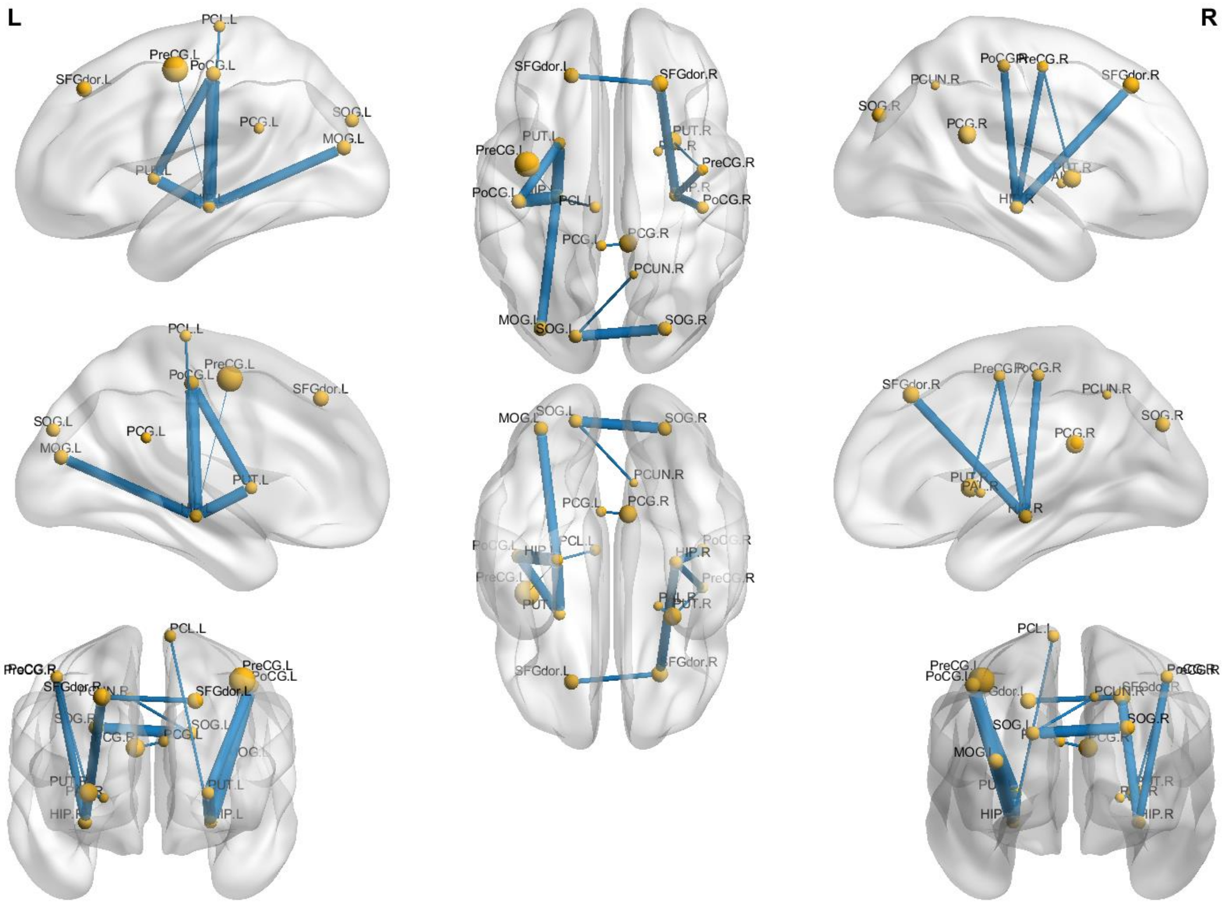

Supplemental figure 4. Top 15 discriminative brain functional connections on early prediction of cognitive deficits.

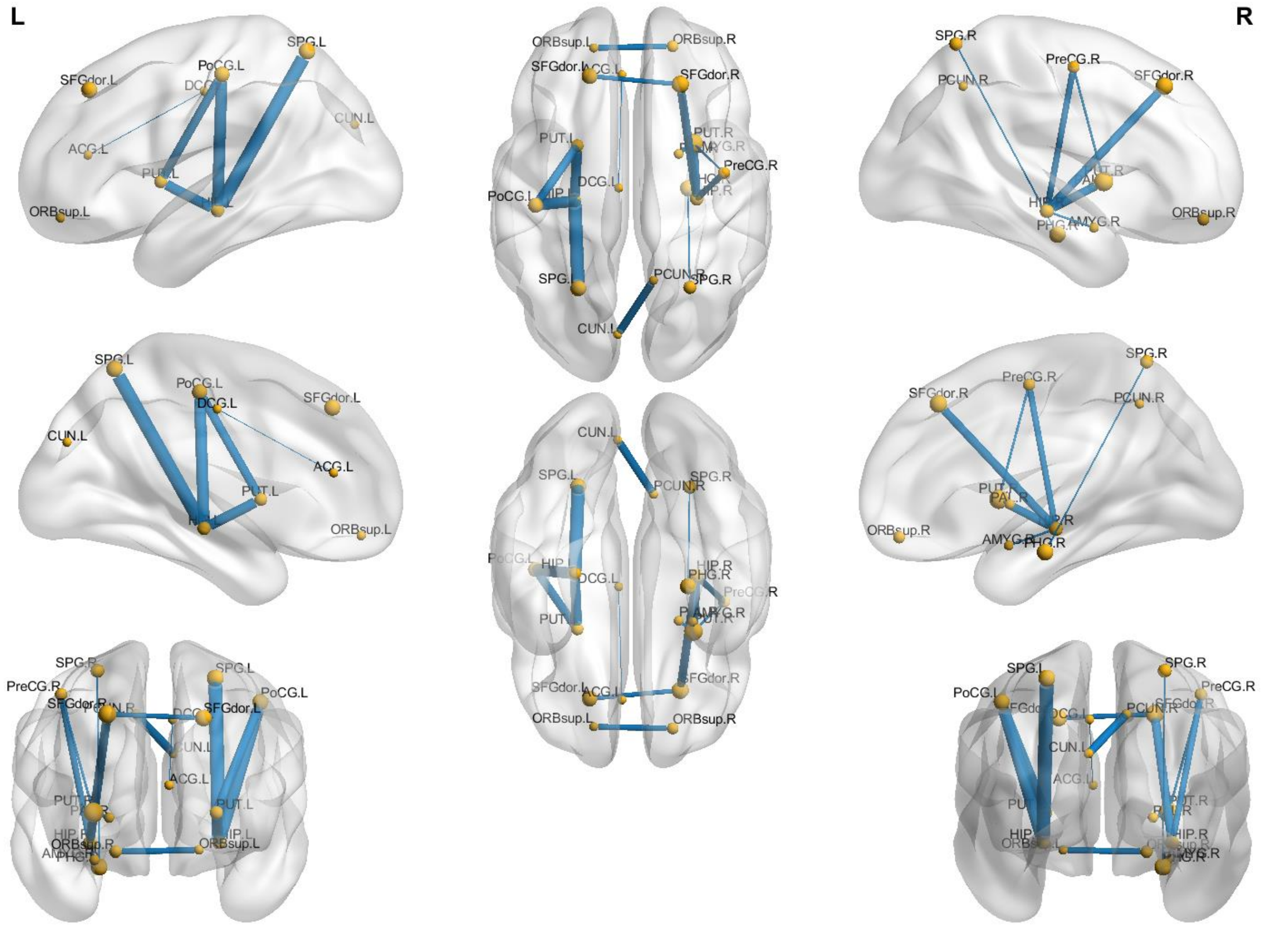

Supplemental figure 5. Top 15 discriminative brain functional connections on early prediction of language deficits.

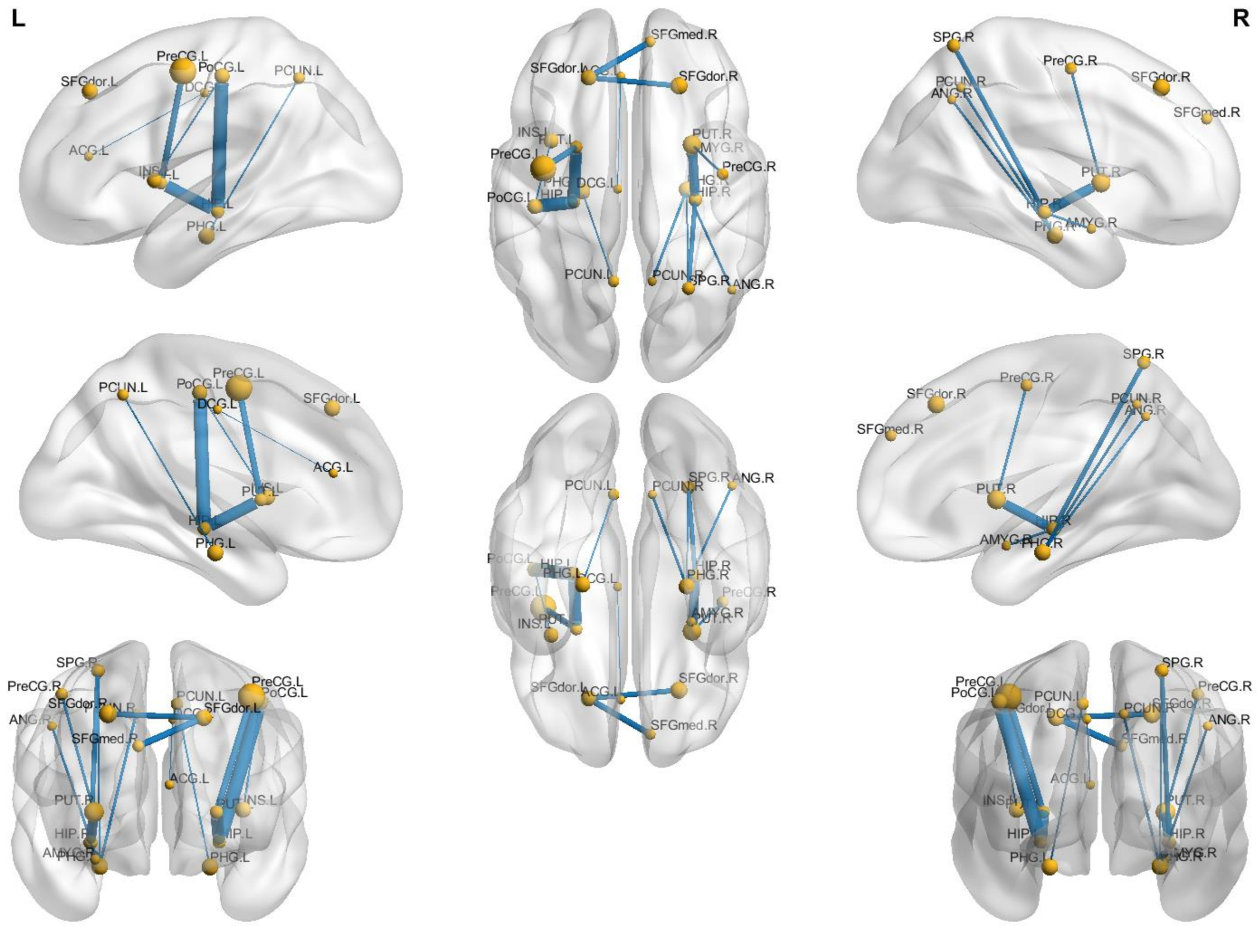

Supplemental figure 6. Top 15 discriminative brain functional connections on early prediction of motor deficits.

2 Supplementary Tables

Supplemental Table 1. Brain MRI scanning parameters.

	Cohort I	Cohort II
**Anatomical MRI**	TR = 8300 ms, TE = 166 ms, FA = 90°, and resolution 1.0 × 1.0 × 1.0 mm^3	TR = 9500 ms, TE = 147 ms, FA = 150°, resolution 0.93 × 0.93 × 1.0 mm^3
**rs-fMRI**	TR = 1195 ms, TE = 45 ms, FA = 55°, resolution 2.5 × 2.5 × 2.5 mm^3, and 400 frames	TR = 3000/1000 ms, TE = 35/30 ms, FA = 90°/50°, and resolution 2.8 × 2.8 × 3.0 mm^3, and 300 frames
**DTI**	TR = 7000 ms, TE = 88 ms, field of view = 160 × 160 mm^2, resolution = 2.0 × 2.0 × 2.0 mm^3, number of slices = 54, noncolinear diffusion-weighted directions (b = 800 s/mm^2)=36, and sensitivity encoding factor = 2	TR = 7500/9553/3996 ms, TE = 77/89/104 ms, field of view = 160 × 160 mm^2, resolution = 2.0 × 2.0 × 2.0 mm^3, number of slices = 76, noncolinear diffusion-weighted directions (b = 800/2000 s/mm^2)=64, and sensitivity encoding factor = 2

TR: repetition time; TE: echo time (TE); FA: flip angle

Supplemental Table 2. Full list of 72 clinical features for very preterm infants in the cohort.

Category	Index	Variable name	Range/Proportion	Note
**Material information**	1	Mothers age (years)	Range:[17,48], Median: 32	
	2	Gravida	Range:[1,8], Median: 2	
	3	Parity	Range:[1,5], Median: 2	
	4	Marital status	52 (72.7%)	Married
	5	Highest education level	Range:[2,7], Median: 6	1. <7th grade,2. 7th to 9th grade,3. 10th to 12th grade,4. High School degree,5. Partial college,6. College degree,7. Graduate degree,8. Unknown
	6	Maternal annual income	Range:[1,9], Median: 5	1. less than $10k, 2. $10k- $19k, 3. $20k - $29k, 4. $30k - $39k, 5. $40k - $59k, 6. $60k - $79k, 7. $80k - $99k, 8. More than 100k. 9. Prefer not to answer
	7	Maternal smoking status	27 (35.5%)	Yes
	8	Street drugs during pregnancy	6 (8.3%)	Yes
	9	Narcotics during pregnancy	65 (90.3%)	No
**Pregnancy complications**	10	Multiple birth	23 (31.9%)	Yes
	11	Diabetes insulin	2 (2.7%)	Yes
	12	Hypertension	22 (30.5%)	Yes
	13	Hyperthyroidism	7 (9.7%)	Yes
	14	Antepartum hemorrhage	15 (20.8%)	Yes
	15	Chorioamnionitis	6 (18.2%)	Yes
	16	Placental pathology	34 (47.2%)	Yes

	17	Fertility treatment	16 (22.2%)	Yes
**Labor and delivery**	18	Rupture of membrane	28 (38.9%)	Yes
	19	Steroids	70 (97.2%)	Yes
	20	Magnesium	47 (65.3%)	Yes
	21	Antibiotics	58 (80.6%)	Yes
	22	Delivery mode	18 (25.0%)	Yes
	23	Contractions	21 (29.2%)	Yes
	24	Infections history	21 (29.2%)	Yes
	25	Fever during labor	17 (23.6%)	Yes
**Neonatal information at birth**	26	Birth hospital	Range:[1,4], Median: 2	1. OSU,2. RMH,3. SA, 4. Other
	27	Clamping delay >30 sec	21 (29.2%)	
	28	Umbilical cord milked prior to clamping	63 (87.5%)	
	29	Infant sex	39 (54.2%)	Male
	30	Ethnic categories	71 (98.6%)	Not Hispanic or Latino
	31	Racial categories	Range:[1,6], Median: 2	1. Black,2. White,3. American Indian or Alaskan Native,4. Asian,5. Native Hawaiian or other Pacific Islander,6. More Than One Race,7. Unknown or Not Reported
	32	Gestational age at birth (total weeks)	Range:[23,31], Median: 28	
	33	Gestational age at birth (total days)	Range:[168,229], Median: 215	
	34	Oxygen support at birth	68 (94.4%)	Yes
	35	Birth weight (grams)	Range:[510,2340], Median: 1055	
	36	Birth length (cm)	Range:[28,43.5], Median: 37.5	

	37	Head circumference at birth (cm)	Range:[19.5,31.5], Median: 25.7	
**Neonatal information at term follow-up**	38	Referral to child protective services	65 (90.3%)	No
**Medical history**	39	Oxygen or positive pressure support	5 (6.9%)	No
	40	Surfactant	39 (54.2%)	No
	41	Pneumothorax	67 (93%)	No
	42	Pulmonary hemorrhage	71 (98.6%)	No
	43	Steroids for BPD/CLD	67 (93%)	No
	44	Total number of days on oxygen therapy	Range:[0,114], Median: 43.5	
	45	Total number of days on CPAP	Range:[0,85], Median: 35.5	
	46	Ventilation therapy	Range:[0,93], Median: 0	
	47	Total days on HFOV	Range:[0,17], Median: 0	
	48	Respiratory support type at 36-week	15 (20.5%)	No
	49	Pulmonary hypertension history	70 (97.2%)	No
	50	Patent ductus arteriosus history	56 (77.8%)	No
	51	Chest compressions history	69 (95.8%)	No
	52	Indomethacin history for prophylaxis	59 (81.9%)	No

	53	Seizure history	70 (97.2%)	No
	54	Cranial sonograms history before 35 weeks PMA	10 (13.8%)	No
	55	Cranial sonograms history at or after 35 weeks PMA	20 (27.8%)	No
	56	PMA at scan (total weeks)	Range:[39.3,42], Median: 40.0	
	57	PMA at scan (total days)	Range:[256,301], Median: 287	
	58	Early onset septicemia/bacteremia	70 (97.2%)	No
	59	Late onset culture positive septicemia/bacteremia	63 (87.5%)	No
	60	Meningitis	71 (98.6%)	No
	61	Parenteral alimentation (total days)	Range:[5,80], Median: 14	
	62	Breast milk in the first 28 days	70 (97.2%)	No
	63	Necrotizing enterocolitis	66 (91.7%)	No
	64	GI surgery that resulted in short gut	70 (97.2%)	No
	65	PRBC transfusions	39 (54.1%)	No
	66	Caffeine for apnea/neuroprotection	9 (12.5%)	No
	67	Iron supplementation	2 (2.7%)	No
	68	Hearing screen	11 (15.3%)	No
	69	Retinopathy of prematurity exam	8 (11.1%)	No
	70	Retinopathy of prematurity status	Range:[1,3], Median: 3	1 = Determined, favorable in both eyes, 2 = Determined, severe ROP in either eye, 3 = Undetermined ROP status in either

				eye (and neither had “severe ROP”)
	71	Major surgery	63 (87.5%)	No
	72	Syndromes and/or major malformations	69 (95.8%)	No
